# Venous Thromboembolic Events in Adult Trauma Patients Receiving Balanced Hemostatic Resuscitation (BHR): An Analysis of Their Incidence, Predictors, and Associated Mortality Rates at a Level 1 Trauma Center

**DOI:** 10.7759/cureus.59679

**Published:** 2024-05-05

**Authors:** Dia R Halalmeh, Antonia Vrana, Phillip Jenkins, James A Cranford, Kristoffer Wong, Dean Kristl, Leo Mercer, Marc D Moisi, Gul R Sachwani-Daswani

**Affiliations:** 1 Trauma and Acute Care Surgery, Hurley Medical Center, Flint, USA; 2 Radiation Oncology, Detroit Medical Center, Detroit, USA; 3 Emergency Medicine, University of Michigan, Ann Arbor, USA; 4 Surgery, Texas Tech University Health Science Center, Lubbock, USA; 5 Neurosurgery, Hurley Medical Center, Flint, USA

**Keywords:** predictors, tranexamic acid, balanced resuscitation, massive transfusion, mortality, trauma, venous thromboembolism

## Abstract

Background and objective

Studies assessing the incidence of venous thromboembolic (VTE) events in the setting of massive balanced transfusions and/or tranexamic acid (TXA) infusion have yielded varied outcomes. In light of this, we conducted this study to examine the incidence of VTEs in trauma patients requiring blood products, and to identify the risk factors for VTE and mortality in this population.

Methods

We performed a retrospective analysis of trauma patients admitted to our level 1 trauma center from January 2013 to September 2023. Clinical characteristics were compared between patients who developed VTE and those who did not. A regression analysis of potential variables associated with the development of VTEs and mortality was performed.

Results

Among 1305 patients (mean age: 42.4 ± 18.8 years) receiving blood products within the initial 24 hours, 4.3% (56 patients) developed a VTE. Patients with VTE experienced prolonged ICU and hospital stays and ventilation duration (p<0.001). They were also noted to have delayed initiation of VTE prophylaxis (104.2 vs. 50.3 hours, p<.001). Prolonged ventilation >7 days was the sole significant factor associated with VTE in multivariate regression analysis [odds ratio (OR): 6.2, p=0.004]. Early TXA administration (within four hours) showed a higher association with VTE than TXA within 24 hours (OR: 2.1, p=0.07 vs. OR 1.6, p=0.22). Massive transfusion was found to increase VTE risk (OR: 2.65, p<0.001). Severe head and neck (OR: 6.0, p=0.002) and chest (OR: 3.8, p=0.01) injuries were key predictors of mortality, while TXA was not significantly associated with mortality in the multivariate model.

Conclusions

Our study revealed an elevated risk of VTE in patients requiring massive transfusion protocol (MTP, ≥6 units). Early TXA administration was neither associated with increased VTE risk in MTP patients nor increased mortality risk. Strategies directed at reducing the risk of VTE in massively transfused patients while maintaining the survival benefits of balanced resuscitation and TXA need to be devised.

## Introduction

Venous thromboembolism (VTE) is a complication of trauma stemming from several factors, including immobilization, surgical intervention, endothelial trauma-releasing tissue factor, and the associated inflammatory prothrombotic state [1]. The incidence of VTE in trauma patients ranges from 3.8% to 70% in various studies in the literature [2,3]. This variation in reported incidence among studies is partly attributed to the differences in diagnostic modality, trauma severity, and patient characteristics and comorbidities, among other variables [2,4]. Trauma patients can suffer from VTE despite receiving adequate prophylaxis, and it is a major cause of morbidity and mortality in hospitalized trauma patients, with a mortality rate approaching 26% in those who develop pulmonary embolism (PE) [3]. This is further complicated by the additional mortality from hemorrhagic shock, another major cause of death in the setting of trauma [5].

Over the past decade, balanced hemostatic resuscitation (BHR) has replaced the use of aggressive crystalloid resuscitation in trauma patients, as the latter has been found to be associated with hypothermia, acidosis (worsening the performance of coagulation factors), and increased mortality [6]. Coagulopathy resulting from trauma is significantly reduced with the use of BHR [7]. It has been established that using BHR with an ideal ratio of 1:1:1 [packed red blood cells (pRBCs)/plasma/platelets] in trauma management is associated with decreased mortality and improved outcomes [8]. 

The role of tranexamic acid (TXA) in traumatic hemorrhage, including in patients with expected delays in undergoing surgical intervention, has also been studied in randomized clinical trials [9,10]. Recently, evidence of optimized outcomes with the use of TX has been reported in subgroups of patients with severe injury [11]. As with any intervention, BHR and TXA are not devoid of risks. Massive resuscitation and use of antifibrinolytic agents such as TXA certainly interfere with the coagulation pathway. In this study, we aimed to evaluate the incidence of VTEs [i.e., deep vein thrombosis (DVT) and/or PE] in patients receiving BHR, and to characterize the factors associated with VTE in this patient population. We also assessed the effect of adding TXA to BHR on the overall incidence of VTE and mortality. The prognostic indicators of mortality were also evaluated.

## Materials and methods

Selection criteria

**Figure 1:**
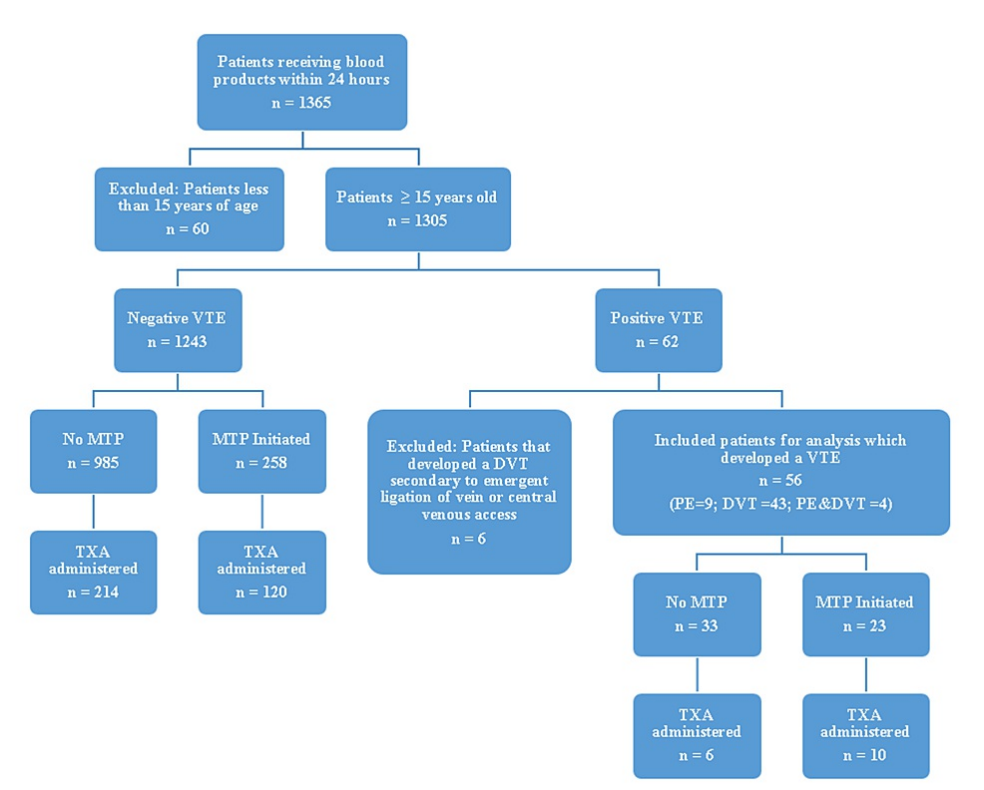
Flowchart depicting the selection process and categorization for subgroup analyses DVT: deep vein thrombosis; MTP: massive transfusion protocol; PE: pulmonary embolism; TXA: tranexamic acid; VTE: venous thromboembolism

After obtaining the IRB approval, a retrospective analysis of trauma patients admitted to our level 1 trauma center was conducted. All adult trauma patients admitted during the period from January 2013 to September 2023 who received any blood product were screened for inclusion. Patients aged less than 15 years were excluded, as we primarily focused on adolescent and adult populations. Patients were then classified into two cohorts based on the development of VTE after admission (Figure 1). Patients with signs and symptoms of DVT and/or PE (based on the modified Well’s criteria )[12,13] were evaluated for VTE and those who tested positive were included in the analysis. Below-knee DVTs were also included. Next, a stratified analysis was performed based on the administration of our institutional massive transfusion protocol (MTP). Institutional MTP was initiated when patients required ≥6 units of pRBCs and/or fresh frozen plasma (FFP). This was considered a BHR procedure since the products were given in a 1:1:1 ratio. TXA administration was also recorded (if given) in each subgroup. Patients who developed DVT secondary to emergent ligation of leg veins or due to central venous access were excluded. The primary endpoint was to examine the overall incidence of VTE in adult trauma patients admitted to the hospital.

Data extraction

The following demographic and other clinical variables were retrieved from the electronic medical records: age (in years), gender, comorbid conditions, BMI, mechanism and type of injuries, abbreviated injury scale (AIS), injury severity score (ISS), procedures performed, type and number of transfusion units over first four hours and 24 hours, history of anticoagulation therapy, number of patients receiving initial and additional TXA infusion, presenting systolic blood pressure (SBP), ICU length of stay (LOS), ventilator duration, hospital LOS, hours of VTE prophylaxis after admission, number of VTEs, days to diagnosis of VTE, and final outcomes.

Statistical analysis

Predefined secondary endpoints included multiple variables that were evaluated and compared between the two main cohorts for statistical significance using the unpaired, two-tailed t-test. To better understand and prevent the development of VTE in trauma patients, we attempted to evaluate the association of these secondary endpoints with VTE. Whether TXA increases mortality in patients with major trauma is an important clinical question. Therefore, we examined prognosticators of mortality to better understand the effect of TXA in the context of other variables, similar to what might be encountered in clinical practice. To control for confounders, binomial univariate and multivariate regression analyses were subsequently performed and compared in the process of examining the predictors of VTE.

The predictive factors compared between groups were as follows: age >40 years; male gender; patients in whom MTP (≥6 units) was initiated within the first four and 24 hours; patients who received MTP ± TXA within the first four and 24 hours; units of IV fluid administered within the first four and 24 hours; hours to initiation of VTE chemoprophylaxis; prophylaxis exceeding 48 hours; ICU LOS; ventilator stay >7 days; ISS (as a continuous variable); severe injury (ISS >24); moderate injury (ISS 10-24); and severe lower extremity injury (AIS ≥4). To determine if the mean difference or ratios were statistically significant, we used the unpaired t-test. Due to the potential presence of multicollinearity between variables, the variance inflation factor (IVF) with a value of or below 10 was considered acceptable.

The alpha level for statistical significance was set at p<0.05. Odds ratios (OR) were estimated to analyze the magnitude of the association between independent and dependent variables. A 95% confidence interval (CI) was also used to correlate with the statistical significance of the measured associations and mean differences. Statistical analyses were performed using SPSS Statistics for Windows, version 26.0 (IBM Corp., Armonk, NY).

## Results

Demographic characteristics and general VTE trends for all trauma patients

From January 2013 to September 2023, a total of 17,093 consecutive trauma patients (34.1% females, 65.9% males) were admitted to our level 1 trauma center. Of those, 6364 (37.2%) patients were older than 15. The mean age of the cohort was 40.5 ± 24.3 years (range: 15-105 years). The average ISS was 9.36. Approximately 9.5% of patients had ISS greater than 24 (i.e., severe injury), whereas 23.8% had ISS between 10 and 24 (moderate injury). The average annual incidence rate of VTE was 0.9%.

Trauma patients receiving blood products

Of 17,093 patients, 1305 received blood products within the first 24 hours of presentation. Sixty patients below the age of 15 years were excluded from the analysis (Figure 1). Of the remaining patients (1305), 62 developed VTE. Six patients were excluded because the VTE was induced by a procedure (e.g., leg vein ligation, central venous access). DVT was the most common form of VTE (76.8%) followed by PE (16.0%). Only four patients had DVT and PE concurrently. Demographic and clinical characteristics of patients who received any blood product (1305) are summarized in Table 1. Adult males formed the bulk of this population, with an average age of 42.4 ± 18.8 years. Based on ISS, 43.5% of those patients had severe injury (ISS ≥25). Fourteen (14%) patients were on anticoagulants/antiplatelets before the injury, most commonly aspirin (7.7%). The mean IV fluid quantity administered during the first 24 hours was 3.2 ± 3.1 L. Patients who received any amount of blood products had a blood product ratio of 1:1:0.6. In contrast, those who received balanced MTP had a ratio of ~1:1:1. In total, 26.8% of patients received TXA. The average time to initiate VTE chemoprophylaxis after admission was 53.6 hours. The overall mortality rate was 14.7%.

**Table 1:** Characteristics of trauma patients (age >15 years) who received blood products (n=1305) *Six patients were excluded because their VTE developed secondary to emergent ligation of leg veins or due to central venous access AIS: abbreviated injury scale; DVT: deep vein thromboembolism; ICU: intensive care unit; ISS: injury severity score; IV: intravenous; LOS: length of stay; MTP: massive transfusion protocol; PE: pulmonary embolism; SBP: systolic blood pressure; SD standard deviation; TXA: tranexamic acid; VTE: venous thromboembolism

Variable	Value
Age, years, mean ± SD	42.4 ± 18.8
Gender, n (%)	
Male	987 (75.9%)
Female	318 (24.3%)
Anatomical region of injury, n (%)	
Upper extremity injury	502 (38.5%)
Lower extremity injury	720 (55.2%)
Head and neck injury	550 (42.1%)
Facial injury	396 (30.3%)
Abdominal injury	652 (50.0%)
Chest injury	718 (55.0%)
Traumatic brain injury	379 (29.0%)
Spine injury	668 (51.2%)
Pelvic injury	227 (17.4%)
ISS, mean ± SD	22.3 ± 14.4
≤9 (mild)	269 (20.6%)
10-24 (moderate)	382 (29.3%)
≥25 (severe)	568 (43.5%)
AIS, mean ± SD	
Head and neck	3.0 ± 1.4
Face	1.4 ± 0.5
Chest	3.1 ± 1.0
Abdomen	2.9 ± 1.2
Upper extremity	1.8 ± 0.7
Lower extremity	2.6 ± 0.9
External	1.2 ± 0.9
Initial SBP (mean ± SD)	112.8 ± 41.9
IV fluid, mmHg, mean ± SD	
First 4 hours	1.6 ± 1.7 L
First 24 hours	3.2 ± 3.1 L
Blood products, units, mean	
First 4 hours	
RBC	3.5
FFP	3.4
PLT	1.5
First 24 hours	
RBC	4.5
FFP	4.2
PLT	2.6
Patients receiving MTP (≥6 units), n (%)	281 (21.5%)
Blood products within the first 24 hours in patients receiving MTP	
RBC	12.2
FFP	11.7
PLT	9.6
Patients receiving TXA, n (%)	350 (26.8%)
Average time to start VTE chemoprophylaxis after admission, hours	53.6
Patients who developed VTE, n (%)	62 (4.7%)^*^
DVT	43
PE	9
DVT and PE	4
Hospital LOS, days, mean (range)	13.0 (1-313)
ICU stay, days	11.6 (1-153)
Ventilator duration, days	7.3 (1-89)
Outcome, n (%)	
Rehabilitation	296 (22.7%)
Discharged home	535 (41.0%)
Discharged to a skilled facility	121 (9.3%)
Discharged against medical advice	14 (1.7%)
Mortality	191 (14.7%)
ISS ≥25	152 (79.6%)
ISS 10-24	36 (18.7%)
ISS ≤9	3 (1.5%)

Patients who received any blood product were initially divided into two groups according to the development of VTE. Table 2 summarizes the statistical differences concerning select variables between the two cohorts. There was a statistically significantly greater number of patients receiving MTP in the non-VTE groups compared to the VTE group (p=0.0014). In addition, mean pRBC in the first four and 24 hours, mean FFP in the first 24 hours, ICU LOS, ventilator duration, and hospital LOS, and hours to initiation of VTE prophylaxis were statistically significantly higher in the VTE group (p<0.05). These factors were subsequently analyzed for association with VTE in the logistic regression analysis.

**Table 2:** Demographic and clinical characteristics of trauma patients (age >15 years) requiring administration of blood products (n=1305) *Patients who developed a DVT secondary to emergent ligation of vein or central venous access were excluded (n=6) AIS: abbreviated injury scale; DVT: deep vein thromboembolism; FFP: fresh frozen plasma; ICU: intensive care unit; ISS: injury severity score; IV: intravenous; LOS: length of stay; MTP: massive transfusion protocol; PE: pulmonary embolism; pRBC: packed red blood cells; SBP: systolic blood pressure; SD standard deviation; TXA: tranexamic acid; VTE: venous thromboembolism

Variable	Total (n=1305)	Negative VTE (n=1243)	Positive VTE (n=56)^*^	P-value
Age, years, mean ± SD	42.4 ± 18.8	42.3 ± 18.9	43.9 ± 17.1	0.52
Male gender, n (%)	981 (75.1%)	935 (75.2%)	46 (82.1%)	0.55
Patients receiving MTP, ≥6 units, n (%)	281 (21.5%)	258 (20.7%)	23 (41.0%)	0.0014
Patients receiving TXA bolus, n (%)	350 (26.8%)	334 (26.8%)	16 (28.5%)	0.81
pRBC in first 4 hours, mean ± SD	3.4 ± 4.4	3.3 ± 4.3	4.7 ± 3.8	0.025
pRBC in first 24 hours, mean ± SD	4.4 ± 6.0	4.3 ± 5.9	6.3 ± 5.2	0.015
FFP in first 24 hours, mean ± SD	4.2 ± 6.0	4.0 ± 5.9	5.9 ± 5.0	0.017
Platelets in first 24 hours, mean ± SD	2.5 ± 7.2	2.5 ± 7.2	3.7 ± 6.1	0.19
ICU LOS (n=566), mean ± SD	11.6 ± 13.9	10.7 ± 13.4	24.5 ± 16.8	<0.001
Ventilator duration, days, mean ± SD	7.2 ± 9.7	6.5 ± 8.6	18.3 ± 16.7	<0.001
Hospital LOS, days, mean ± SD	13.0 ± 16.8	12.2 ± 16.4	29.8 ± 16.5	<0.001
Hours to initiation of VTE prophylaxis, mean ± SD	53.6± 60.4	50.3 ± 51.7	104.2 ± 126.8	<0.001
Overall ISS, mean ± SD	22.3 ± 14.4	22.1 ± 14.9	25.3 ± 12.8	0.10
Severe injury (ISS >24), mean ± SD	35.5 ± 11.8	35.6 ± 12.0	36.0 ± 7.5	0.86
Moderate injury (ISS 10-24), mean ± SD	16.5 ± 4.3	16.5 ± 4.4	16.1 ±3.8	0.66
Mild injury (ISS <10), mean ± SD	6.0 ± 3.0	5.9 ± 3.0	7.5 ± 2.2	0.17
AIS, mean ± SD				
Lower extremity injury	2.6 ± 0.9	2.6 ± 0.9	2.8 ± 0.7	0.19
Head and neck injury	3.0 ± 1.4	3.0 ± 1.4	3.1 ± 1.4	0.86
Abdominal injury	2.88 ± 1.1	2.89 ± 1.1	2.82 ± 1.1	0.75
Chest injury	3.13 ± 1.0	3.1 ± 1.0	3.2 ± 0.8	0.45
History of VTE, n (%)	15 (0.1%)	15 (0.1%)	0	0.41
Type of injury, n (%)				
Vertebral column and/or spinal cord	178 (13.6%)	171 (13.7%)	7 (12.5%)	0.84
Pelvic fractures	223 (17.0%)	215 (17.3%)	8 (14.3%)	0.59
Long bone fractures	275 (21.0%)	261 (20.9%)	14 (25.0%)	0.52
Required operative intervention due to injury	326 (24.9%)	316 (25.4%)	10 (17.8%)	0.26

**Figure 2:**
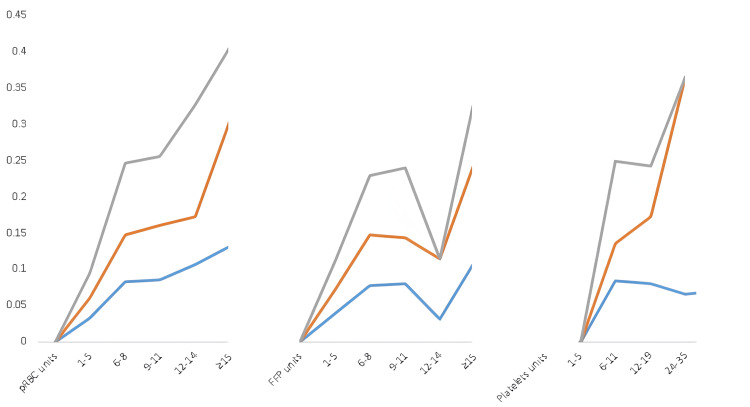
Incidence of VTE with the increasing number of units of the three main blood products (pRBC, FFP, and PLT) Grey line: incidence of VTE (n%). Orange line: incidence of VTE in patients who received TXA. Blue line: incidence of VTE in patients without TXA FFP: fresh frozen plasma; PLT: platelets; pRBC: packed red blood cells; VTE: venous thromboembolism

To better characterize the influence of each blood product in VTE development, patients who received blood products were further analyzed according to the amount (in liters) of each blood product (unit category) and TXA administration (Table 3). The incidence of VTE was not statistically significantly different between TXA and non-TXA groups for all unit categories. However, pairwise analysis based on unit category only (regardless of TXA administration) showed that the incidence of VTE was statistically significantly higher in those who received >15 units of pRBC (p<0.001), 12-14 units of FFP (p<0.003), or 24-36 units of platelets (p<0.02) compared to other categories (Figure 2).

**Table 3:** Incidence of VTE in trauma patients based on units of blood products and TXA administration within the first 24 hours (n=1305) ^*^P-value for the difference in VTE incidence between TXA and non-TXA patients. ^#^Pairwise comparison analysis: each category was compared against every other unit category in pairs pRBC: packed red blood cells; TXA: tranexamic acid; VTE: venous thromboembolism

	Incidence of VTE, n (%)	Incidence of VTE in patients who received TXA, n (%)	Incidence of VTE in patients without TXA, n (%)	P-value*	Pairwise comparison analysis^#^	P-value
Overall	56 (4.3%)	18/353 (5.0%)	44/952 (4.6%)	0.72		<0.001
pRBC units						
1-5	34/1019 (3.3%)	6/220 (2.7%)	28/799 (3.5%)	0.57	>15 units pRBC	
6-8	11/132 (8.3%)	4/62 (6.4%)	7/70 (10%)	0.48		
9-11	5/58 (8.6%)	2/27 (7.4%)	3/31 (9.6%)	0.76		
12-14	3/28 (10.7%)	1/15 (6.6%)	2/13 (15.4%)	0.48		
≥15	9/68 (13.2%)	5/29 (17.2%)	4/39 (10.2%)	0.43		
≥6	28/286 (9.7%)	12/133 (9.0%)	16/153 (10.4%)	0.69		
FFP units						<0.003
1-5	33/844 (3.9%)	6/178 (3.3%)	27/666 (4.0%)	0.68	12-14 units FFP	
6-8	10/129 (7.7%)	4/57 (7.0%)	6/72 (8.3%)	0.78		
9-11	5/62 (8.0%)	2/31 (6.4%)	3/31 (9.6%)	0.65		
12-14	1/32 (3.1%)	1/12 (8.3%)	0/20 (0%)	0.19		
≥15	7/62 (11.2%)	4/29 (13.8%)	3/33 (9.0%)	0.58		
≥6	23/285 (8.0%)	11/129 (8.5%)	12/156 (7.8%)	0.80		
Platelets units						
1-5	0	-	-	-	24-36 units of platelets	<0.02
6-11	14/166 (8.4%)	4/78 (5.1%)	10/88 (11.4%)	0.16		
12-19	6/75 (8.0%)	3/32 (9.3%)	3/43 (6.9%)	0.71		
24-35	3/18 (16.6%)	3/10 (30%)	0/8 (0%)	0.12		
≥36	1/14 (7.1%)	0/5 (0%)	1/9 (11.1%)	0.45		
≥6	24/273 (8.7%)	10/125 (8.0%)	14/148 (9.4%)	0.68		

Multivariate and univariate regression analysis of the predictors of VTE development and mortality

Several factors were assessed to evaluate any potential significant association with the development of VTE (Table 4). Compared to the null model, the multivariate logistic regression model was statistically significant (X^2^=36.9; p<0.001). In addition, the multivariate regression model was 91% accurate in predicting the outcomes of interest, namely VTE and mortality. The relationship between various variables and the two major outcomes, development of VTE and death, was evaluated using two separate models.

**Table 4:** Univariate and multivariate logistic regression analysis of factors associated with the development of VTE in trauma patients receiving blood products AIS: abbreviated injury scale; CI: confidence interval; ICU: intensive care unit; ISS: injury severity score; LOS: length of stay; MTP: massive transfusion protocol; OR: odds ratio; TXA: tranexamic acid; VTE: venous thromboembolism

Variable	Univariate analysis		Multivariate analysis	
	OR [95% CI]	P-value	OR [95% CI]	P-value
MTP within the first 4 hours	2.58 [1.4, 4.6]	0.002	1.7 [0.2, 10.4]	0.55
MTP within the first 24 hours	2.65 [1.5, 4.6]	<0.001	1.7 [0.3, 10.3]	0.53
MTP + TXA within the first 4 hours	2.1 [0.9, 4.9]	0.07	0.59 [0.07, 4.9]	0.62
MTP + TXA within the first 24 hours	1.61 [0.7, 3.5]	0.22	0.43 [0.11, 1.6]	0.22
Age >40 years	1.11[0.6, 1.9]	0.70	1.02 [0.3, 2.7]	0.95
Male gender	1.60 [0.77, 3.31]	0.20	2.5 [0.5, 12.0]	0.22
Hours to initiation of VTE chemoprophylaxis	1.008 [1.005, 1.019]	<0.001	1.0 [0.99, 1.01]	0.10
Prophylaxis more than 48 hours	1.9 [1.08-3.33]	0.024	0.85 [0.27, 2.74]	0.87
IV fluid within the first 4 hours	1.3 [1.1, 1.5]	0.002	1.2 [0.8, 2.0]	0.24
IV fluid within the first 24 hours	1.09 [1.02, 1.17]	0.01	0.9 [0.7, 1.1]	0.74
ICU LOS	1.03 [1.02, 1.05]	<0.001	0.98 [0.92, 1.03]	0.50
Ventilator duration >7 days	8.3 [3.9, 17.7]	<0.001	6.2 [1.79, 21.48]	0.004
Hospital LOS	1.03 [1.02, 1.04]	<0.001	1.02 [0.97, 1.07]	0.31
Severe injury (ISS >24)	1.72 [0.99, 3.00]	0.052	0.8 [1.7, 3.6]	0.78
Severe lower extremity injury (AIS ≥4)	0.5 [0.1, 2.3]	0.39	0.23 [0.01, 4.89]	0.35

**Figure 3:**
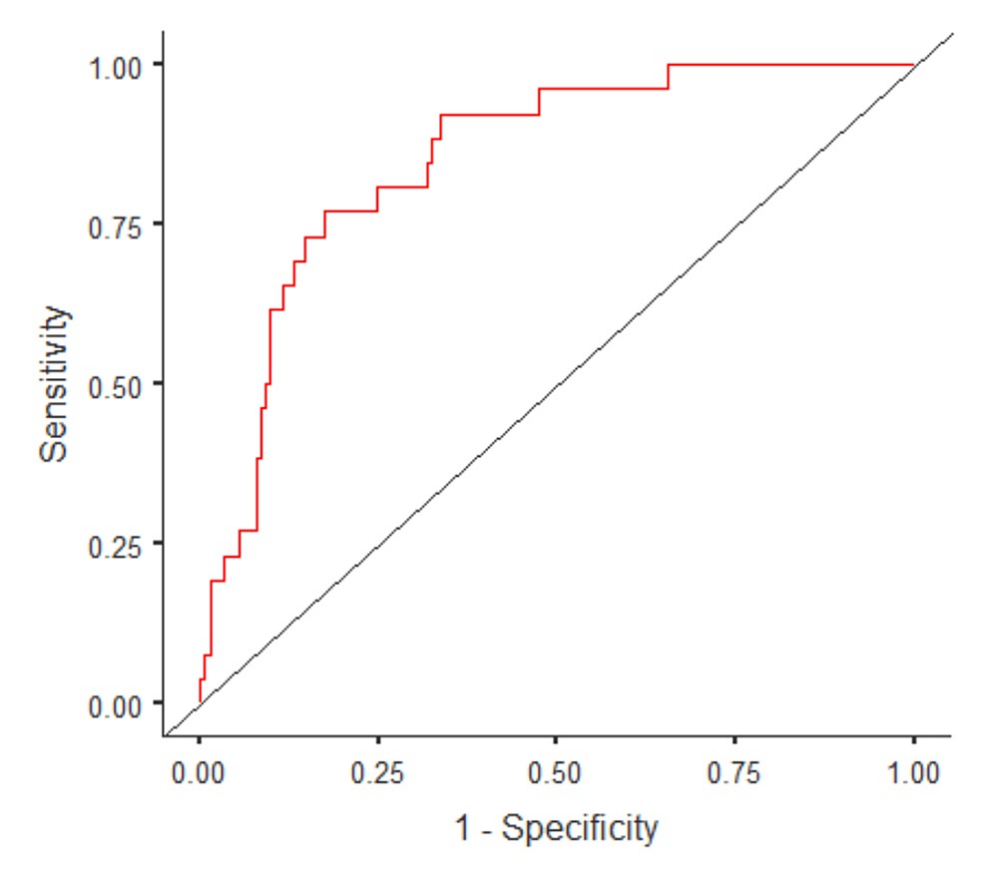
Receiver operating characteristic curve (ROC) showing the predictive power of the multivariate logistic regression model Area under the curve (AUC): 0.84. Accuracy: 0.91

Based on the univariate regression analysis, MTP within the first four and 24 hours (OR: 2.58, p=0.002; OR 2.65, p<0.001, respectively), prophylaxis exceeding 48 hours (OR: 1.9, p=0.024), and ventilator duration >7 days (OR: 8.3, p<0.001) were statistically significant risk factors for VTE in trauma patients receiving blood products. However, the use of TXA in MTP patients was not statistically significantly associated with VTE development (p=0.07). On multivariate analysis, the strongest and the only risk factor for VTE was ventilation for more than seven days (OR: 6.2, p=0.004). Predictive measures were calculated using the receiver operating characteristic curve (ROC) (Figure 3).

Predictors for mortality were also evaluated (Table 5). On univariate analysis, the factors significantly associated with death were severe injury (ISS ≥24), severe head and neck injury (AIS≥4), severe chest injury (AIS ≥4), and MTP with and without TXA. However, on multivariate regression, the most significant prognostic indicator was severe head and neck injury (OR: 6.0, p=0.002), followed by severe chest injury (OR: 3.8, p=0.001). Although the odds of mortality were ~4 times higher in those who received MTP with TXA within the first 4 hours, this did not reach statistical significance (OR: 4.4, p=0.08). The mortality rate was 24.1% in patients who received TXA and 21.6% in those who did not, with no statistically significant difference (p=0.39).

**Table 5:** Prognostic factors of mortality in trauma patients receiving blood products AIS: Abbreviated injury scale; CI: confidence interval; ISS: injury severity score; MTP: massive transfusion protocol; OR: odds ratio; TXA: tranexamic acid

Variable	Univariate analysis		Multivariate analysis	
	OR [95% CI]	P-value	OR [95% CI]	P-value
Severe injury (ISS >24)	11.5 [7.2, 18.3]	<0.001	2.8 [0.4, 17.1]	0.25
Age >40 years	1.01 [0.7, 1.4]	0.93	2.0 [0.7, 5.7]	0.18
Severe head and neck injury (AIS ≥4)	7.4 [4.1, 13.5]	<0.001	6.0 [1.9, 18.7]	0.002
Severe chest injury (AIS ≥4)	5.07 [3.0, 8.3]	<0.001	3.8 [1.3, 10.6]	0.01
Severe lower extremity injury (AIS ≥4)	1.01 [0.4, 2.3]	0.96	1.12 [0.2, 4.7]	0.85
MTP within the first 4 hours	3.7 [2.3, 5.9]	<0.001	4.9 [0.6, 41.7]	0.13
MTP + TXA within the first 4 hours	4.4 [2.4, 8.1]	<0.001	5.0 [0.8, 31.5]	0.08
MTP within the first 24 hours	3.3 [2.2, 5.1]	<0.001	2.6 [0.7, 8.8]	0.11
MTP + TXA within the first 24 hours	4.05 [2.3, 6.9]	<0.001	1.5 [0.24, 10.03]	0.62

## Discussion

Trauma-induced coagulopathy (TIC) occurs in approximately 30% of patients with major trauma [14]. In TIC, a brief hypercoagulable state initially occurs, which is immediately followed by a hypocoagulability, and eventually transitions back to a hypercoagulable state [14]. This rapid coagulation status shift is believed to result from a sequence triggered by trauma-induced tissue injury, blood loss, and physiological responses like hypothermia and acidosis [6]. In response to severe injury, there is typically significant thrombin generation. Insufficient upregulation of thrombin is associated with high mortality and increased transfusion requirements [15].

Notably, hypocoagulable states after trauma are more prevalent in patients with higher ISS and are linked to higher mortality rates [16]. In our study, severe injury (ISS>24) emerged as the most powerful independent prognostic factor for death (OR: 11.5, p<0.001) on the univariate analysis. The effect of hypothermia, acidosis, and hypotension triad along with ISS was evaluated by Cosgriff et al. in their prospective analysis of 58 patients receiving massive transfusions (>10 units). They demonstrated a substantial rise (up to 49%) in the likelihood of developing life-threatening coagulopathy when ISS exceeded 25 and temperature was below 34 °C [16]. Similar findings were observed in our study, where 80.8% of fatalities had ISS ≥25 (Table 1). This can be attributed in part to prolonged hypoperfusion, and the release of substantial tissue factor amounts, triggering thromboembolic events and end-organ damage, which are common in severe injuries.

The underlying mechanisms driving the transition from the hypocoagulable to the later hypercoagulable states are multifactorial and include endothelial injury, circulatory stasis, platelet activation, decreased levels of endogenous anticoagulants, and impaired fibrinolysis [17]. Trauma has been shown to reduce levels of natural anticoagulant agents like activated C protein (APC), protein S, and antithrombin III (ATIII) [1,2]. The propensity for delayed hypercoagulability in trauma patients raises concerns about VTE risk in this population. Enhanced thrombin generation rate has been recognized as an independent predictor of trauma-related VTE, suggesting its potential as a risk assessment marker [18]. The initial hypocoagulable phase in TIC is exacerbated by resuscitation strategies reliant solely on large crystalloid volumes without clotting factor administration [19]. BHR has emerged as a counteractive strategy, involving early administration of pRBC, plasma, and platelets, often in a 1:1:1 ratio. This administration ratio correlates with favorable outcomes and reduced mortality [8,20]. In our study, the institutional MTP ensured an approximate 1:1:1 administration ratio for all trauma patients receiving blood products (Table 1).

The introduction of BHR and TXA in trauma care has prompted concerns about an elevated risk of VTE in trauma patients who already possess a predisposition to hypercoagulability. Notably, the administration of massive transfusion alone has been observed to influence the subsequent development of VTE in trauma patients [21]. Previous research indicates a dose-dependent relationship, with the risk of VTE rising proportionately to the number of pRBCs transfused [21]. In our analysis, a similar dose-dependent association was identified, particularly in cases where pRBC transfusions exceeded 15 units, exhibiting the highest VTE incidence compared to other categories (p<0.001), as evident from pairwise comparison analysis (Table 3). An increasing number of units of other blood products such as FFP and PLT were also associated with greater VTE incidence, albeit not as consistently as observed with pRBC transfusions (Figure 2).

In the present study, a higher proportion of patients who received MTP was found in the VTE group compared to the non-VTE group (41.0% vs. 20.7%, p=0.0014). Univariate regression analysis showed that MTP administration within the first four and 24 hours was a statistically significant risk factor for VTE (OR: 2.58 p=0.002, OR: 2.65 p=<0.001). The statistically significant association between MTP within the initial four and 24 hours and VTE development suggests that MTP independently increases the risk of VTE, irrespective of the timing of administration. Furthermore, pairwise comparison analysis revealed a notably elevated incidence of VTE, specifically in individuals who received >15 units of pRBC (p<0.001), 12-14 units of FFP (p<0.003), or 24-36 units of platelets (p<0.02). These findings reinforce the findings of prior studies, indicating an increased VTE risk in individuals receiving massive transfusions.

Studies exploring the association between TXA and VTE have yielded mixed results. Some authors found that TXA was associated with a threefold increase in the odds of VTE [22], while others did not find any significant association [23,24]. A recent meta-analysis spanning diverse medical disciplines revealed that patients treated with TXA did not exhibit an elevated risk of thromboembolic complications [25]. McDuffie et al. reported an increase in PE rates following BHR implementation, potentially influenced by other factors like the concurrent administration of TXA. However, no clear independent association was found [26]. The MATTERS trials demonstrated that MT patients who received TXA had a higher risk of VTE compared to those who did not, a finding echoed by Myers et al. and Adair et al. [22,27]. Adair et al. showed that TXA was less predictive of VTE than the mechanism of injury and injury type [28].

In our study, there was no statistically significant difference in the proportion of patients receiving TXA between the VTE and non-VTE groups (28.5% vs. 26.8%, p=0.81). On univariate analysis, the odds of VTE were 2.1 times higher in MTP patients who received TXA than in the non-TXA group, although this did not reach statistical significance (p=0.07). However, our observations suggested that the timing of TXA administration might influence VTE occurrence. Administering MTP with TXA within the initial 24-hour period did not exhibit a statistically significant association with VTE development (OR: 1.61, p=0.22). However, when administered within the first four hours, MTP with TXA demonstrated a notable trend toward significance (OR: 2.1, p=0.07). These findings imply that administering MTP with TXA within the first four hours might pose a potential risk for VTE, suggesting a time-dependent aspect to their effects, although this did not reach statistical significance.

We systematically assessed multiple factors for their correlation with two primary outcomes: the occurrence of VTE and mortality in trauma patients. Multivariable regression models revealed prolonged ventilation (>7 days) to be the strongest and most consistently observed risk factor for VTE in trauma patients receiving blood products (OR: 6.2, p=0.004). When controlling for other factors in the univariate analysis, initiating prophylaxis exceeding 48 hours and initiating MTP within the first four and 24 hours were independently associated with VTE development (Table 4). This aligns with the current evidence in the literature. Prabhakaran et al. examined numerous predictors for VTE and found that ICU and hospital LOS were comparable between VTE and non-VTE groups [29]. Our study found no statistically significant association between these two variables and VTE incidence in multivariate analysis, suggesting limited clinical utility of this information, especially in severely injured patients who typically require intensive monitoring and life-saving interventions, services typically available only in the ICU. On the other hand, the length of mechanical ventilation was established as a risk factor for VTE in several previous studies [3,4,29]. Notably, MTP with or without TXA did not exhibit a statistically significant association with VTE according to multivariate regression analysis (Table 4). This emphasizes the crucial role of a balanced ratio of blood products in mitigating trauma-induced coagulopathy and improving outcomes as seen in previous studies [8].

TXA has emerged as an effective intervention for trauma patients. Findings from the CRASH-2 trial as well as the MATTERS I and MATTERS II studies have demonstrated the efficacy of TXA in reducing bleeding and mortality following trauma [9,27]. However, the timing of TXA administration is critical, as early administration within one hour of trauma has shown significant mortality reduction [27]. Conversely, delayed administration may exacerbate bleeding or other complications [27]. This timing effect, illustrated in the CRASH-2 trial, was attributed to the development of disseminated intravascular coagulation (DIC) in the later stages of trauma or increased incidences of hypothermia and acidosis in patients with delayed arrival at the hospital, factors that may diminish TXA efficacy [9]. When TXA is administered to patients requiring massive transfusion, a similar reduction in mortality has been observed. According to MATTERS trials, individuals receiving MTP along with TXA experienced a mortality rate of 14.4%, compared to 28.1% for those receiving MTP alone [9,27].

It should be noted that the existing evidence regarding the benefits of TXA in trauma patients primarily relies on differences in mortality rates between patient groups. In our study, although the mortality rate was higher in patients who received TXA (24.1%) compared to those who did not (21.6%), this difference did not reach statistical significance (p=0.39). To gain a better understanding of the relationship between TXA and patient outcomes, we conducted a logistic analysis to explore whether the mortality benefits remained consistent across various predictive models, reflective of clinical practice. In contrast to the MATTERS study findings, our univariate analysis revealed higher odds of mortality in patients receiving MTP with and without TXA (OR: 4.05 and 3.3, p<0.001). However, upon incorporating other variables into the multivariate analysis (Table 5), these associations lost significance, likely due to the impact of injury patterns on mortality and morbidity. Notably, severe head and neck injuries, as well as severe chest injuries, emerged as statistically significant predictors of mortality (OR: 6.0 and OR 3.8, p<0.05).

We advocate for the use of TXA in this patient population, particularly within the initial four hours, owing to its clinically observed survival benefit and its potential to reduce VTE incidence (although the related findings in our study were statistically nonsignificant: OR 0.59, p=0.62). This recommendation is underscored by the urgent need to manage hemorrhage in severely injured patients, where failure to undertake necessary measures could result in fatal exsanguination. Consequently, prospective and more extensive multicenter controlled studies may be essential to further delineate the genuine mortality-related benefits of TXA administration in massively transfused patients.

Massive transfusion and TXA administration are frequently observed in patients with higher ISS. Higher ISS values have been previously identified as an independent risk factor for VTE [30]. It is imperative to discern whether the increased VTE risk in trauma patients receiving MT, TXA, or MT + TXA is a consequence of the transfusion itself or the typically higher ISS within the population undergoing these interventions. In our study, the ISS, whether considered as a continuous or categorical variable, was not a statistically significant risk factor for VTE (Table 4). The presence and complexity of additional confounding factors need to be acknowledged when conducting these analyses. Adjusting for these variables is often essential to validate any meaningful associations identified through regression analysis. For instance, a propensity-matched analysis was performed by Sara et al. and they found a significant increase in mortality following TXA administration in the univariate unadjusted model. However, after accounting for variables reported in the literature, the impact of TXA on mortality became nonsignificant [22].

In our series, various regression analysis models were employed to explore the interdependencies among study variables and their impact on VTE development. During univariate regression, TXA was notably associated with mortality (OR: 4.4 and 4.04, p<0.001). However, this association lost significance when other variables were considered in the multivariate analysis (Table 5). This suggests that multiple factors exert varying effects on predicting survival in severely injured patients undergoing massive transfusion.

Limitations

Certain limitations are intrinsic to all retrospective analyses and must be acknowledged. Numerous univariate and multivariate regression models were employed to mitigate the potential impact of missing information on the computed associations. Since the pattern of missing data was completely random, and due to the ability to obtain statistical significance (indicating sufficient power), the effect of loss of information was minimal. Caution is warranted when interpreting factors and predictors, especially those demonstrating marginal significance. These findings indicate associations rather than causation. The absence of a control group poses challenges in terms of establishing causality.

Considering the mixed evidence regarding the effect of TXA use in conjunction with MTP on VTE incidence, more prospective and randomized controlled studies across multiple centers are required to elucidate the relationship between these variables. Additionally, it is pertinent to note that the study was conducted at a level 1 trauma center, with distinct demographic and clinical variables compared to tertiary or level 2 and 3 centers. Given that TXA administration is largely based on the treating physician's discretion and despite discernible trends suggesting an association with VTE, this is unlikely to alter providers' practices given the absence of unambiguous and consistent evidence in the literature.

## Conclusions

The implementation of BHR and TXA has raised concerns regarding a potential increased risk of VTE, particularly in patients receiving MTP. It is crucial to note that VTE in trauma patients is multifaceted, and the effect of TXA is heavily influenced by patient- and trauma-related factors. Our single-center study has identified trends that indicate a possible time-dependent aspect related to the combined administration of massive transfusion and TXA concerning VTE risk. Regardless of the VTE risk, urgent life-saving hemostatic interventions, including TXA administration, should take precedence and should not be delayed. Severe head, neck, and chest injuries were strongly associated with mortality. Among MT trauma patients, prolonged ventilation exceeding seven days was the most significant risk factor for VTE. Recognizing predictors of VTE and mortality in trauma patients is vital for enhancing decision-making processes and avoiding unnecessary interventions while managing these patients. While our retrospective analysis provides valuable insights, further prospective and randomized controlled studies involving multiple centers are imperative to broaden our understanding of these intricate interactions and optimize strategies for the management of trauma patients.
